# Safety and efficacy of atezolizumab plus bevacizumab in elderly patients with hepatocellular carcinoma: A multicenter analysis

**DOI:** 10.1002/cam4.4763

**Published:** 2022-04-19

**Authors:** Toshifumi Tada, Takashi Kumada, Atsushi Hiraoka, Masashi Hirooka, Kazuya Kariyama, Joji Tani, Masanori Atsukawa, Koichi Takaguchi, Ei Itobayashi, Shinya Fukunishi, Kunihiko Tsuji, Toru Ishikawa, Kazuto Tajiri, Hironori Ochi, Satoshi Yasuda, Hidenori Toyoda, Chikara Ogawa, Takashi Nishimura, Takeshi Hatanaka, Satoru Kakizaki, Noritomo Shimada, Kazuhito Kawata, Takaaki Tanaka, Hideko Ohama, Kazuhiro Nouso, Asahiro Morishita, Akemi Tsutsui, Takuya Nagano, Norio Itokawa, Tomomi Okubo, Taeang Arai, Michitaka Imai, Atsushi Naganuma, Yohei Koizumi, Shinichiro Nakamura, Kouji Joko, Hiroko Iijima, Yoichi Hiasa, the Real‐life Practice Experts for HCC (RELPEC) Study Group and the Hepatocellular Carcinoma Experts from 48 clinics in Japan (HCC 48) Group

**Affiliations:** ^1^ Department of Internal Medicine Japanese Red Cross Himeji Hospital Himeji Japan; ^2^ Department of Internal medicine, Division of Gastroenterology and Hepatology Hyogo College of Medicine Nishinomiya Japan; ^3^ Department of Nursing Gifu Kyoritsu University Japan; ^4^ Gastroenterology Center Ehime Prefectural Central Hospital Matsuyama Japan; ^5^ Department of Gastroenterology and Metabology Ehime University Graduate School of Medicine Ehime Japan; ^6^ Department of Gastroenterology Okayama City Hospital Okayama Japan; ^7^ Department of Gastroenterology and Hepatology Kagawa University Kagawa Japan; ^8^ Division of Gastroenterology and Hepatology, Department of Internal Medicine Nippon Medical School Tokyo Japan; ^9^ Department of Hepatology Kagawa Prefectural Central Hospital Takamatsu Japan; ^10^ Department of Gastroenterology Asahi General Hospital Asahi Japan; ^11^ Premier Departmental Research of Medicine Osaka Medical and Pharmaceutical University Osaka Japan; ^12^ Center of Gastroenterology Teine Keijinkai Hospital Sapporo Japan; ^13^ Department of Gastroenterology Saiseikai Niigata Hospital Niigata Japan; ^14^ Department of Gastroenterology Toyama University Hospital Toyama Japan; ^15^ Center for Liver‐Biliary‐Pancreatic Disease Matsuyama Red Cross Hospital Matsuyama Japan; ^16^ Department of Gastroenterology and Hepatology Ogaki Municipal Hospital Japan; ^17^ Department of Gastroenterology Japanese Red Cross Takamatsu Hospital Takamatsu Japan; ^18^ Department of Gastroenterology Gunma Saiseikai Maebashi Hospital Maebashi Japan; ^19^ Department of Clinical Research National Hospital Organization Takasaki General Medical Center Takasaki Japan; ^20^ Division of Gastroenterology and Hepatology Otakanomori Hospital Kashiwa Japan; ^21^ Department of Internal Medicine II Hamamatsu University School of Medicine Hamamatsu Japan; ^22^ Department of Gastroenterology National Hospital Organization Takasaki General Medical Center Takasaki Japan

**Keywords:** adverse events, atezolizumab plus bevacizumab, elderly patient, hepatocellular carcinoma, survival

## Abstract

**Aim:**

The safety and efficacy of atezolizumab plus bevacizumab (Atez/Bev) in elderly patients with unresectable hepatocellular carcinoma (HCC) have not been sufficiently investigated.

**Methods:**

A total of 317 patients with HCC treated with Atez/Bev were studied. We compared the survival and frequency of adverse events in elderly versus non‐elderly patients with HCC who were treated with Atez/Bev using an analysis of inverse probability weighting (IPW).

**Results:**

Univariate analysis adjusted with IPW showed that being elderly is not associated with worse overall or progression‐free survival (hazard ratio [HR], 1.239; 95% confidence interval [CI], 0.640–2.399; *p* = 0.526 and HR, 1.256; 95% CI, 0.871–1.811; *p* = 0.223, respectively). Regarding treatment‐related adverse events, any grade of fatigue, proteinuria, decreased appetite, hypertension, and liver injury occurred in ≥10% of patients. There were no significant differences in treatment‐related adverse events between the elderly and non‐elderly groups. In a subgroup analysis of elderly patients aged 75–79, 80–84, or ≥ 85 years, there were no significant differences in cumulative overall or progression‐free survival among these age groups (*p* = 0.960 and 0.566, respectively). In addition, there were no significant differences in treatment‐related adverse events among these three age groups, except for proteinuria of any grade. In a subgroup analysis of patients treated with Atez/Bev as first‐line systemic therapy, there were no significant differences in cumulative overall or progression‐free survival between the elderly and non‐elderly groups (*p* = 0.728 and 0.805, respectively).

**Conclusions:**

Atez/Bev can be used efficaciously and safely in spite of age in patients with unresectable HCC.

## Graphical abstract

There were no significant differences in cumulative overall or progression‐free survival between elderly and non‐elderly patients treated with atezolizumab plus bevacizumab. There were no significant differences in treatment‐related adverse events between elderly and non‐elderly patients of this cohort. Atezolizumab plus bevacizumab can be used safely and efficaciously regardless of age in patients with unresectable HCC.

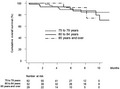

## 1 INTRODUCTION

Primary liver neoplasm is the sixth most commonly detected neoplasm and the third leading cause of cancer‐related death worldwide in 2020, with approximately 906,000 new patients and 830,000 deaths [1]. Hepatocellular carcinoma (HCC) accounts approximately 75%–85% of primary liver cancers [1] and is one of the major health problems in the world. In recent years, there have been remarkable advances in systemic chemotherapy for unresectable HCC. Treatment varies somewhat from country to country, but molecular targeted agents, immune checkpoint inhibitors, and combination therapies with immune checkpoint inhibitors and molecular targeted agents are able to use in clinical practice [2]. In Japan, following introduction of sorafenib [3] as the initial first‐line molecular targeted agent in 2009, approval for lenvatinib [4] as an additional first‐line molecular targeted agent for unresectable HCC was granted in 2018. As second‐line molecular targeted agents, regorafenib [5] was approved in 2017, ramucirumab [6] in 2019, and cabozantinib [7] in 2020. This expansion of treatment options has improved the outcome of patients with unresectable HCC [8].

Recently, the aging of the population has been remarkable in a lot of developing countries. In Europe, in 2011, the life expectancy exceeded 80 years, and it has continued to increase from that time [9]. In the United States, seniors aged 65 and older make up 13% of the population, and those aged 85 and older comprised 1.8% [10]. In Japan, aging is more remarkable. As a result, in Japan, the rate of age‐adjusted HCC‐related death has increased in recent decades [11].

Atezolizumab plus bevacizumab (Atez/Bev) was established as first‐line systemic chemotherapy for unresectable HCC [12]. This systemic therapy consists of an immune checkpoint inhibitor and a molecular targeted agent [12]. In a phase 3 trial, this combination therapy had superior treatment efficacy for improving outcome in patients with advanced HCC than sorafenib as a first‐line molecular targeted agent [12]. However, the efficacy and safety of Atez/Bev in elderly patients with advanced HCC have been insufficiently researched in real‐world settings.

In this study, we studied the survival and frequency of treatment‐related adverse events in elderly versus non‐elderly patients with unresectable HCC treated with Atez/Bev. In addition, we evaluated outcome by an analysis of inverse probability weighting (IPW) to control for biases and confounding factors in observational researches [13].

## 2 MATERIALS AND METHODS

### 2.1 Patients

This research was a retrospective investigation of dataset records according to the Guidelines for Clinical Research issued by the Ministry of Health and Welfare of Japan. The protocol of study was approved by the institutional ethics committee of Ehime Prefectural Central Hospital (IRB # 30–66) (UMIN‐000043219) and each participating institution. All procedures were conducted based on the Declaration of Helsinki. Written informed consent including based on opt‐out method was obtained from all patients.

We enrolled 317 patients with unresectable HCC who received Atez/Bev between September 2020 and October 2021 at 20 institutions in Japan (Japanese Red Cross Himeji Hospital [*n* = 45], Kagawa University Hospital [*n* = 35], Nippon Medical School Hospital Group (Chiba Hokusoh Hospital and Sendagi Hospital) [*n* = 29], Ehime Prefectural Central Hospital [*n* = 27], Asahi General Hospital [*n* = 27], Ehime University Hospital [*n* = 22], Gunma Saiseikai Maebashi Hospital [*n* = 15], Osaka Medical and Pharmaceutical University Hospital [*n* = 15], Okayama City Hospital [*n* = 15], Ogaki Municipal Hospital [*n* = 14], Otakanomori Hospital [*n* = 12], Japanese Red Cross Takamatsu Hospital [*n* = 12], Kagawa Prefectural Central Hospital [*n* = 12], Toyama University Hospital [*n* = 9], Takasaki General Medical Center [*n* = 7], Hamamatsu University Hospital [*n* = 5], Matsuyama Red Cross Hospital [*n* = 4], Teine Keijinkai Hospital [*n* = 4], Saiseikai Niigata Hospital [*n* = 4], and Hyogo College of Medicine College Hospital [*n* = 4]).

In this study, we defined “elderly patients” as patients aged ≥75 years. Regarding to HCC etiology, patients positive for hepatitis B virus surface antigen were diagnosed to have HCC due to the hepatitis B virus infection; those positive for hepatitis C virus antibodies were diagnosed to have HCC due to the hepatitis C virus infection. Hepatic function was assessed using the Child‐Pugh classification system [14].

The date when Atez/Bev therapy began was defined as the start of follow‐up. The end of follow‐up was defined as the date of the final visit for patients who remained alive and the date of death for patients who died during follow‐up.

### 2.2 Diagnosis and treatment of HCC

In this study, HCC was diagnosed according to increases in α‐fetoprotein levels as well as dynamic computed tomography, dynamic magnetic resonance imaging, contrast‐enhanced ultrasonography with perflubutane, or pathological findings [15, 16]. HCC stage was diagnosed according to the Barcelona Clinic Liver Cancer (BCLC) classification system [17].

The most suitable treatment methods for HCC in each patient were determined through discussion among hepatologists, surgeons, oncologists, and radiologists at each hospital according to Japanese practice guidelines for HCC [18, 19]. In this study, Atez/Bev therapy was indicated for unresectable hepatocellular carcinoma. However, even with BCLC stage A, this therapy was indicated when cardiopulmonary function precluded hepatic resection or when radiofrequency/microwave ablation therapy was difficult due to the surrounding organs of the liver or intrahepatic vascular effects. In addition, in patients with BCLC stage D, the attending physician considered the risks and benefits of Atez/Bev therapy for these patients. Then, if it was determined that there was a benefit to this therapy, these patients were offered this treatment with fully informed consent. Patients with a known history of auto‐immune disease were not treated with Atez/Bev.

### 2.3 Atez/Bev treatment and assessment of adverse event

After written informed consent was obtained from the study patient, Atez/Bev therapy, consisting of intravenous 1200 mg of Atez plus 15 mg/kg of body weight of Bev, was administered every 3 weeks [12]. Atez/Bev was discontinued if clinical tumor progression or any unacceptable or severe treatment‐related adverse events occurred.

Treatment‐related adverse events were evaluated by the National Cancer Institute Common Terminology Criteria for Adverse Events, version 5.0 [20]. The clinical guidelines for Atez/Bev therapy created by the manufacturer were used for discontinuation or reduction of each component agent if a treatment‐related adverse event occurred. If Atez/Bev therapy was stopped, the attending physician for each patient made decisions on the administration of another treatment according to Japanese practice guidelines for HCC [18, 19].

### 2.4 Therapeutic response evaluation

Physicians at each hospital assessed tumors using dynamic computed tomography, dynamic magnetic resonance imaging, or contrast‐enhanced ultrasonography with perflubutane at 6 weeks after starting Atez/Bev, based on the Response Evaluation Criteria in Solid Tumors (version 1.1) [21]. Contrast‐enhanced ultrasonography was used for imaging evaluation in patients with contrast allergy on computed tomography or magnetic resonance imaging, or renal dysfunction.

### 2.5 Statistical analysis

In this study, continuous variables are expressed as medians (interquartile range). The Mann–Whitney U‐test or Kruskal–Wallis test was used for in comparison with continuous variables. The χ^2^ test or Fisher's exact test was used for in comparison with categorical variables. Actuarial analysis of cumulative overall survival and progression‐free survival was carried out using the Kaplan–Meier approach, and differences were assessed by the log‐rank test.

In the present study, we used IPW to the Kaplan–Meier analysis and Cox proportional hazards models analyses to adjust for potential imbalances between groups with elderly and non‐elderly patients in overall survival and progression‐free survival. Probabilities for elderly and non‐elderly patients (propensities) were calculated by multiple logistic regression analysis with the following covariates, which were considered likely to be linked with prognosis in patients with advanced HCC: gender, Eastern Cooperative Oncology Group performance status (ECOG‐PS), HCC etiology, Child‐Pugh classification, BCLC classification, and history of systematic therapy [11, 12, 22]. All these variables were included, regardless of statistical significance. Inverse probability weight was specified as 1/(propensity score) for the group of elderly and 1/(1 − propensity score) for the group of non‐elderly. IPW according to the average treatment effect weights method was performed [23].

Statistical significance was defined as *p* < 0.05. Statistical analyses were carried out with EZR version 1.53 (Saitama Medical Center, Jichi Medical University, Saitama, Japan), which is a graphical user interface for R (The R Foundation for Statistical Computing, Vienna, Austria) [24]. More precisely, it is a modified version of the R commander designed to add statistical functions frequently used in biostatistics.

## 3 RESULTS

### 3.1 Patient characteristics

The characteristics of the 317 study patients at baseline are listed in Table 1. There were 59 (18.6%) females and 258 (81.4%) males. The median age was 74.0 (68.0–80.0) years. There were 143 (45.1%) elderly and 174 (54.9%) non‐elderly patients. There were 299 (94.3%) patients with Child‐Pugh class A disease, 17 (5.4%) patients with class B disease, and 1 (0.3%) patient with class C disease. At the start of Atez/Bev therapy, there were 26 (8.2%), 129 (40.7%), 158 (49.8%), and 4 (1.3%) patients with BCLC stage A, B, C, and D HCC, respectively. The Median follow‐up was 5.7 (3.0–8.5) months.

**TABLE 1:** Characteristics of the study patients

	Overall(*n* = 317)	Elderly patients(*n* = 143)	Non‐elderly patients(*n* = 174)	*p*value
Age^a^ (years)	74.0 (68.0–80.0)	80.0 (77.0–83.5)	68.5 (63.0–72.0)	<0.001
Sex (female/male)	59/258	35/108	24/150	0.020
ECOG‐PS (0/1/2/3/4)	252/54/9/1/1	100/37/5/1/0	152/17/4/0/1	<0.001
Body mass index (kg/m^2^)	22.9 (20.7–25.3)	23.2 (20.8–25.0)	22.7 (20.6–25.7)	0.698
Etiology of HCC (hepatitis B/C/non‐B, non‐C)	55/105/157	13/52/78	42/53/79	0.002
Aspartate aminotransferase (IU/L)	38 (27–56)	38 (28–52)	37 (27–58)	0.486
Alanine aminotransferase (IU/L)	27 (19–39)	26 (18–38)	29 (20–41)	0.038
Albumin (g/dL)^a^	3.7 (3.3–4.1)	3.7 (3.4–4.0)	3.7 (3.3–4.1)	0.826
Total bilirubin (mg/dL)^a^	0.7 (0.5–1.0)	0.7 (0.6–0.9)	0.8 (0.5–1.0)	0.303
Platelet count (×10^3^/m^3^)^a^	13.5 (10.6–18.5)	13.0 (10.7–17.1)	14.0 (10.3–19.8)	0.363
Prothrombin time (%)^a^	91 (83–100)	91 (84–100)	92 (81–100)	0.421
Estimated glomerular filtration rate (mL/min/1.73 m^2^)^a^	65.7 (54.0–77.3)	58.8 (48.4–71.5)	72.0 (59.7–85.0)	<0.001
α‐fetoprotein (ng/mL)^a^	39.4 (6.9–591.0)	56.0 (7.5–598.8)	23.7 (6.2–573.3)	0.439
Child‐Pugh class (A/B/C)	299/17/1	139/4/0	160/13/1	0.081
BCLC stage (A/B/C/D)	26/129/158/4	14/69/58/2	12/60/100/2	0.012
Atez/Bev therapy type (first line/other)	179/138	80/63	99/75	0.910
Follow‐up duration^a^ (months)	5.7 (3.0–8.5)	5.4 (2.5–7.9)	5.9 (3.4–9.0)	0.036
Deaths	39	17	22	0.865
Causes of death (liver‐related/ non–liver‐related diseases)	36/3	15/2	21/1	0.570
Propensity score	0.449 (0.319–0.558)	0.509 (0.389–0.618)	0.407 (0.304–0.489)	<0.001
IPW score	0.898 (0.771–1.117)	0.886 (0.729–1.160)	0.926 (0.788–1.160)	0.345

a Data are expressed as medians (interquartile range).

Abbreviations: ECOG‐PS, Eastern Cooperative Oncology Group Performance Status; HCC, hepatocellular carcinoma; BCLC, Barcelona Clinic Liver Cancer; IPW, inverse probability weighting.

The baseline characteristics of the study patients stratified by elderly status are also summarized in Table 1. Age, sex, ECOG‐PS, HCC etiology, alanine aminotransferase level, estimated glomerular filtration rate, BCLC classification, and follow‐up duration were significantly different between elderly and non‐elderly patients.

### 3.2 Cumulative overall survival and cumulative progression‐free survival

**FIGURE 1:**
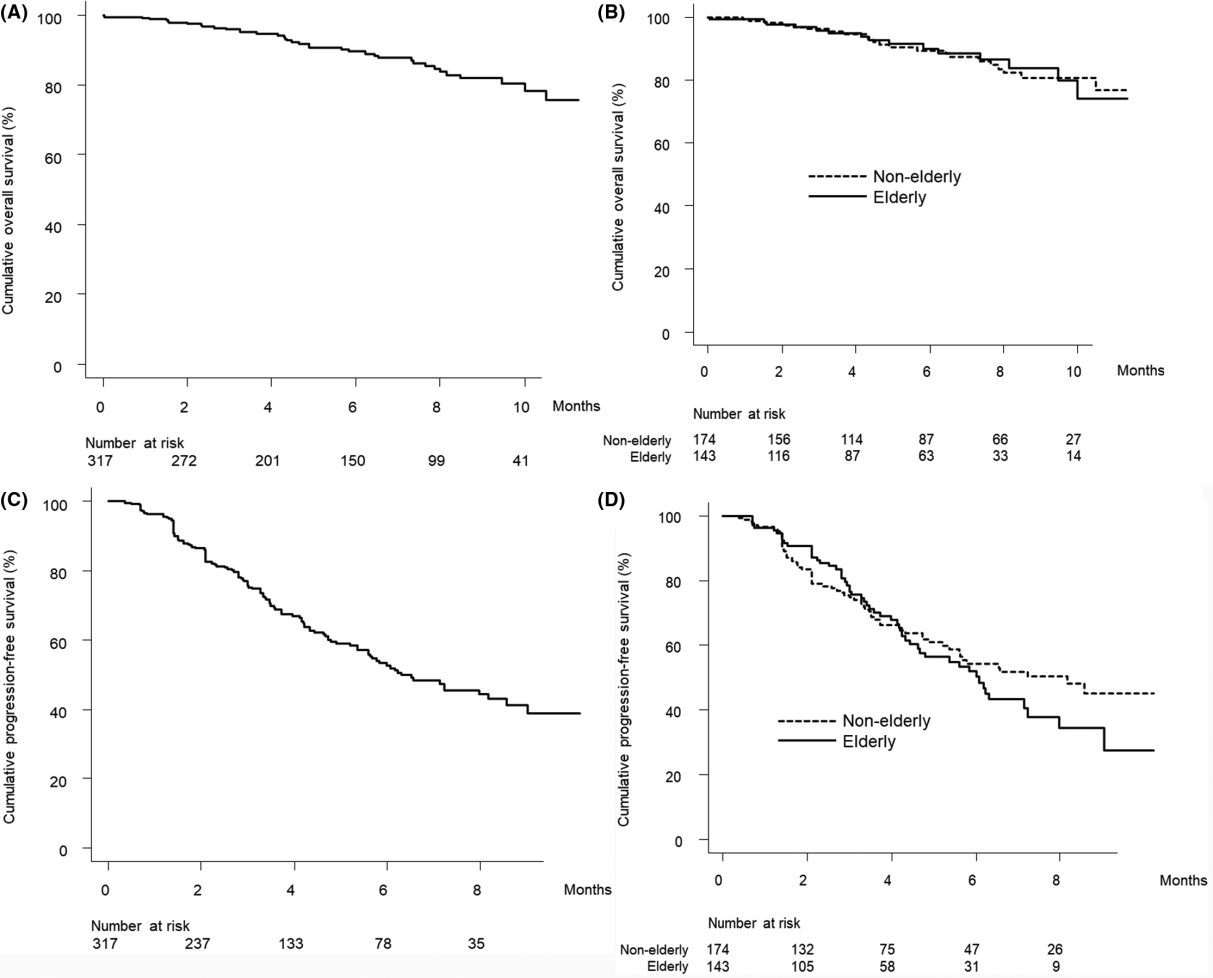
(A) Overall survival. Cumulative overall survival at 3, 6, and 9 months was 96.0%, 89.7%, and 81.9%, respectively. (B) Overall survival stratified by elderly status. Cumulative overall survival at 3, 6, and 9 months was 95.8%, 90.0%, and 83.9% among elderly patients (solid line) and 96.2%, 89.5%, and 80.8% among non‐elderly patients (dotted line), respectively (*p* = 0.735). (C) Progression‐free survival. Cumulative progression‐free survival at 3 and 6 months was 75.6% and 52.7%, respectively. (D) Progression‐free survival stratified by elderly status. Cumulative progression‐free survival at 3 and 6 months was 76.6% and 50.3% among elderly patients (solid line) and 74.8% and 54.2% among non‐elderly patients (dotted line), respectively (*p* = 0.368)

Figure 1A shows the cumulative overall survival curve in this study patients. The cumulative 3‐, 6‐, and 9‐month overall survival rates were 96.0%, 89.7%, and 81.9%, respectively. Figure 1B shows the cumulative overall survival curves stratified by elderly status. There were no significant differences in the cumulative overall survival curves by elderly status (p = 0.735).

Figure 1c shows the cumulative progression‐free survival curve in the study patients. The cumulative 3‐ and 6‐month progression‐free survival rates were 75.6% and 52.7%, respectively. Figure 1d shows the cumulative progression‐free survival curves stratified by elderly status. There were no significant differences in the cumulative progression‐free survival curves by elderly status (*p* = 0.368).

### 3.3 Therapeutic response

The distribution of therapeutic responses is shown in Table 2. In this study, five patients had renal dysfunction and the therapeutic response was determined by contrast‐enhanced ultrasonography. The overall response rate and disease control rate were 26.1% and 81.8%, respectively. There were no significant differences in therapeutic response between the elderly and non‐elderly groups (Table 2).

**TABLE 2:** Therapeutic response

	Overall (*n* = 317)	Elderly patients (*n* = 143)	Non‐elderly patients (*n* = 174)	*p*value
Complete response	9 (3.2%)	5 (4.2%)	4 (2.5%)	0.486
Partial response	64 (22.9%)	31 (26.3%)	33 (20.4%)	
Stable disease	156 (55.7%)	63 (53.4%)	93 (57.4%)	
Progressive disease	51 (18.2%)	19 (16.1%)	32 (19.8%)	
Not evaluated	37	25	12	
Overall response rate	26.1%	30.5%	22.8%	0.169
Disease control rate	81.8%	83.9%	80.2%	0.531

### 3.4 Treatment‐related adverse events

Table 3 lists the Atez/Bev treatment‐related adverse events that occurred in this research cohort. There were no significant differences in treatment‐related adverse events between the elderly and non‐elderly groups.

**TABLE 3:** Adverse events

	Overall (*n* = 317)	Elderly patients (*n* = 143)	Non‐elderly patients (*n* = 174)	*p*value
Liver injury				
Any grade	38 (12.0%)	12 (8.4%)	26 (14.9%)	0.084
Grade ≥3	10 (3.2%)	4 (2.8%)	6 (3.4%)	1.000
Fatigue				
Any grade	70 (22.1%)	33 (23.1%)	37 (21.3%)	0.786
Grade ≥3	3 (0.9%)	2 (1.4%)	1 (0.6%)	0.591
Decreased appetite				
Any grade	63 (19.9%)	32 (22.4%)	31 (17.8%)	0.325
Grade ≥3	11 (3.5%)	7 (4.9%)	4 (2.3%)	0.233
Proteinuria				
Any grade	66 (20.8%)	37 (25.9%)	29 (16.7%)	0.052
Grade ≥3	22 (6.9%)	14 (9.8%)	8 (4.6%)	0.079
Hypertension				
Any grade	50 (15.8%)	18 (12.6%)	32 (18.4%)	0.167
Grade ≥3	12 (3.8%)	8 (5.6%)	4 (2.3%)	0.147
Hypothyroidism				
Any grade	15 (4.7%)	6 (4.2%)	9 (5.2%)	0.794
Grade ≥3	3 (0.9%)	2 (1.4%)	1 (0.6%)	0.591
Fever				
Any grade	25 (7.9%)	8 (5.6%)	17 (9.8%)	0.211
Grade ≥3	4 (1.3%)	1 (0.7%)	3 (1.7%)	0.630
Other				
Any grade	126 (39.7%)	61 (42.7%)	65 (37.4%)	0.358
Grade ≥3	31 (9.8%)	18 (12.6%)	13 (7.5%)	0.134

### 3.5 Analysis using IPWadjustment

**FIGURE 2:**
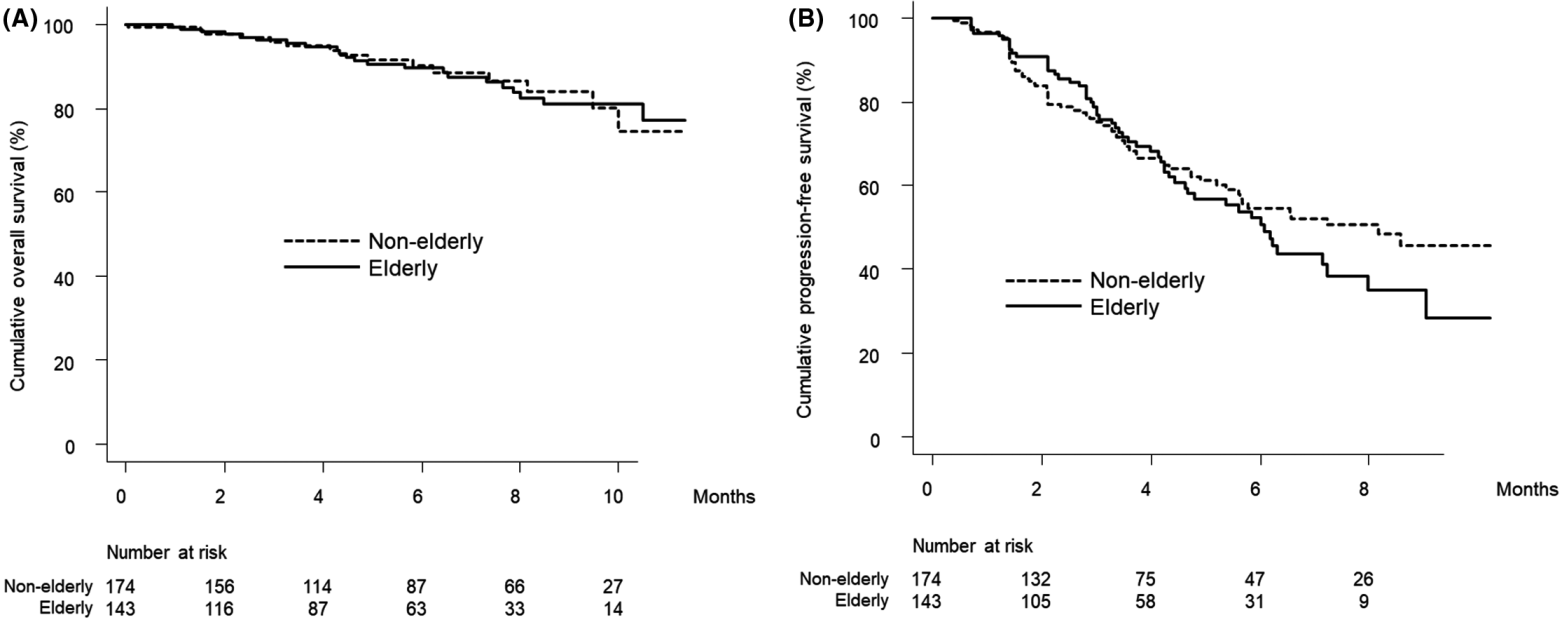
(A) IPW‐adjusted overall survival stratified by elderly status. Cumulative IPW‐adjusted overall survival at 3, 6, and 9 months was 95.9%, 90.0%, and 84.0% among elderly patients (solid line) and 96.2%, 89.5%, and 80.9% among non‐elderly patients (dotted line), respectively. (B) IPW‐adjusted progression‐free survival stratified by elderly status. Cumulative IPW‐adjusted progression‐free survival at 3 and 6 months was 76.8% and 50.6% among elderly patients (solid line) and 75.1% and 54.5% among non‐elderly patients (dotted line), respectively. IPW, inverse probability weighting

Univariate analysis with IPW adjustment showed that being elderly is not associated with worse overall survival in this cohort (hazard ratio [HR], 1.239; 95% confidence interval [CI], 0.640–2.399; *p* = 0.526). Figure 2A shows the IPW‐adjusted cumulative overall survival curves stratified by elderly status.

Univariate analysis with IPW adjustment showed that being elderly is not associated with worse progression‐free survival in this cohort (HR, 1.256; 95% CI, 0.871–1.811; *p* = 0.223). Figure 2B shows the IPW‐adjusted progression‐free cumulative survival curves stratified by elderly status.

### 3.6 Subgroup analysis in elderly patients

**FIGURE 3:**
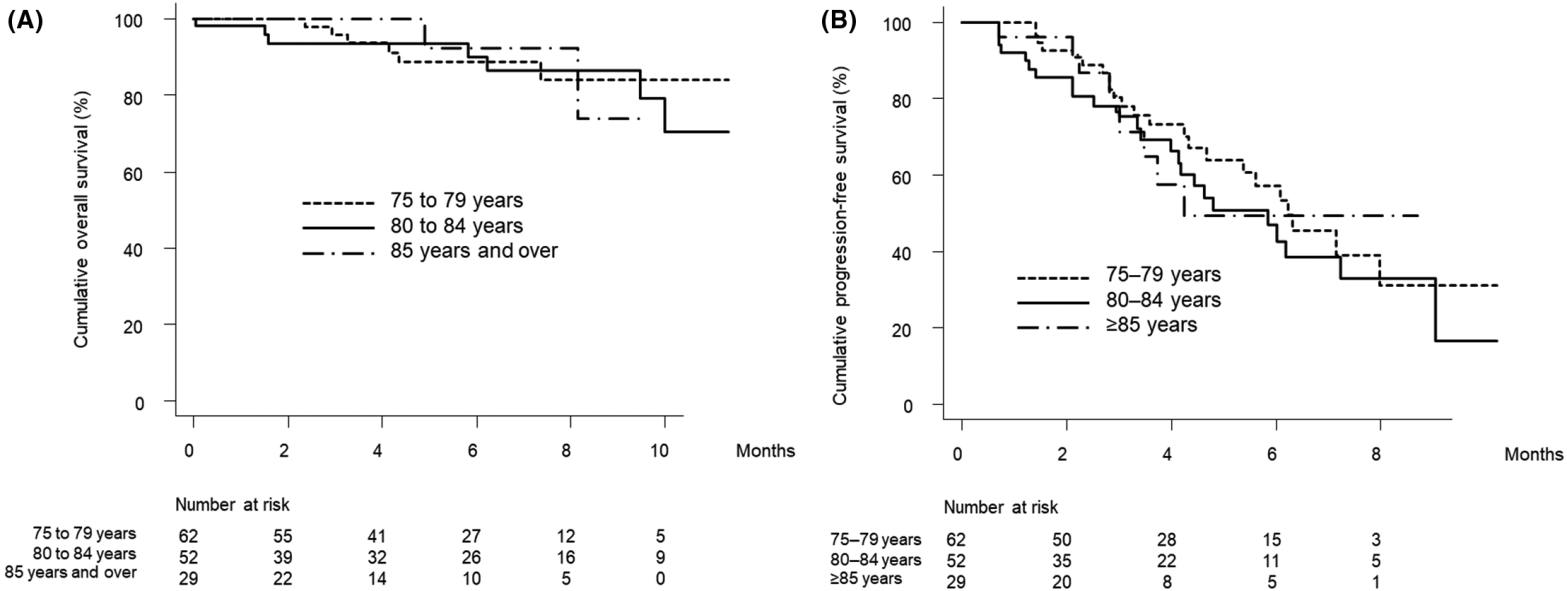
(A) Overall survival stratified by age group in elderly patients. Cumulative overall survival at 3, 6, and 9 months was 96.0%, 88.9%, and 84.2% in elderly patients aged 75–79 years (dotted line), 93.6%, 90.1%, and 86.5% in elderly patients aged 80–84 years (solid line), and 100.0%, 92.3%, and 73.8% in elderly patients aged ≥85 years (dash‐dot‐dash line), respectively (*p* = 0.960). (B) Progression‐free survival stratified by age group in elderly patients. Cumulative progression‐free survival at 3 and 6 months was 80.2% and 57.3% in elderly patients aged 75–79 years (dotted line), 75.3% and 42.7% in elderly patients aged 80–84 years (solid line), and 71.4% and 49.4% in elderly patients aged ≥85 years (dash‐dot‐dash line), respectively (*p* = 0.566).

There were 62, 52, and 29 patients aged 75–79 years, 80–84 years, and ≥ 85 years, respectively. Figure 3A and B show the curves for overall and progression‐free survival stratified by age group. There were no significant differences in the cumulative overall and progression‐free survival curves among these age groups (*p* = 0.960 and 0.566, respectively).

Table 4 shows the distribution of therapeutic responses in elderly patients stratified by age group. There were no significant differences in these therapeutic responses among the three age groups.

**TABLE 4:** Therapeutic response in elderly patients

Age group	75–79 years (*n* = 62)	80–84 years (*n* = 52)	≥85 years (*n* = 29)	*p*value
Complete response	1 (1.9%)	1 (2.3%)	3 (14.3%)	0.066
Partial response	17 (32.1%)	8 (18.2%)	6 (28.6%)	
Stable disease	30 (56.6%)	25 (56.8%)	8 (38.1%)	
Progressive disease	5 (9.4%)	10 (22.7%)	4 (19.0%)	
Not evaluated	9	8	8	
Overall response rate	34.0%	20.5%	42.9%	0.142
Disease control rate	90.6%	77.3%	81.0%	0.191

Table 5 lists the Atez/Bev treatment‐related adverse events that occurred in elderly patients. There were no significant differences in treatment‐related adverse events among the three age groups, except for proteinuria of any grade.

**TABLE 5:** Adverse events in elderly patients

	75–79 years (*n* = 62)	80–84 years (*n* = 52)	≥85 years (*n* = 29)	*p*value
Liver injury				
Any grade	6 (9.7%)	4 (7.7%)	2 (6.9%)	0.577
Grade ≥3	2 (3.2%)	2 (3.8%)	9 (0.0%)	0.581
Fatigue				
Any grade	15 (24.2%)	10 (19.2%)	8 (27.6%)	0.667
Grade ≥3	0 (0.0%)	2 (3.8%)	0 (0.0%)	0.170
Decreased appetite				
Any grade	12 (19.4%)	11 (21.2%)	9 (31.0%)	0.444
Grade ≥3	3 (4.8%)	3 (5.8%)	1 (3.4%)	0.898
Proteinuria				
Any grade	18 (29.0%)	17 (32.7%)	2 (6.9%)	0.030
Grade ≥3	6 (9.7%)	7 (13.5%)	1 (3.4%)	0.347
Hypertension				
Any grade	9 (14.5%)	6 (14.5%)	3 (10.3%)	0.821
Grade ≥3	3 (4.8%)	4 (7.7%)	1 (3.4%)	0.686
Hypothyroidism				
Any grade	3 (4.8%)	2 (3.8%)	1 (3.4%)	0.942
Grade ≥3	1 (1.6%)	1 (1.9%)	0 (0.0%)	0.765
Fever				
Any grade	2 (3.2%)	4 (7.7%)	2 (6.9%)	0.553
Grade ≥3	0 (0.0%)	1 (1.9%)	0 (0.0%)	0.414
Other				
Any grade	27 (43.5%)	23 (44.2%)	11(37.9%)	0.845
Grade ≥3	9 (14.5%)	7 (13.5%)	2 (6.9%)	0.577

### 3.7 Subgroup analysis in patients treated with Atez/Bev as first‐line systemic therapy

In this study, there were 179 patients treated with Atez/Bev as first‐line systematic therapy. The baseline characteristics of this subgroup stratified by elderly status are summarized in Table S1. Age, sex, HCC etiology, estimated glomerular filtration rate, and follow‐up duration were significantly different between elderly and non‐elderly patients.

**FIGURE 4:**
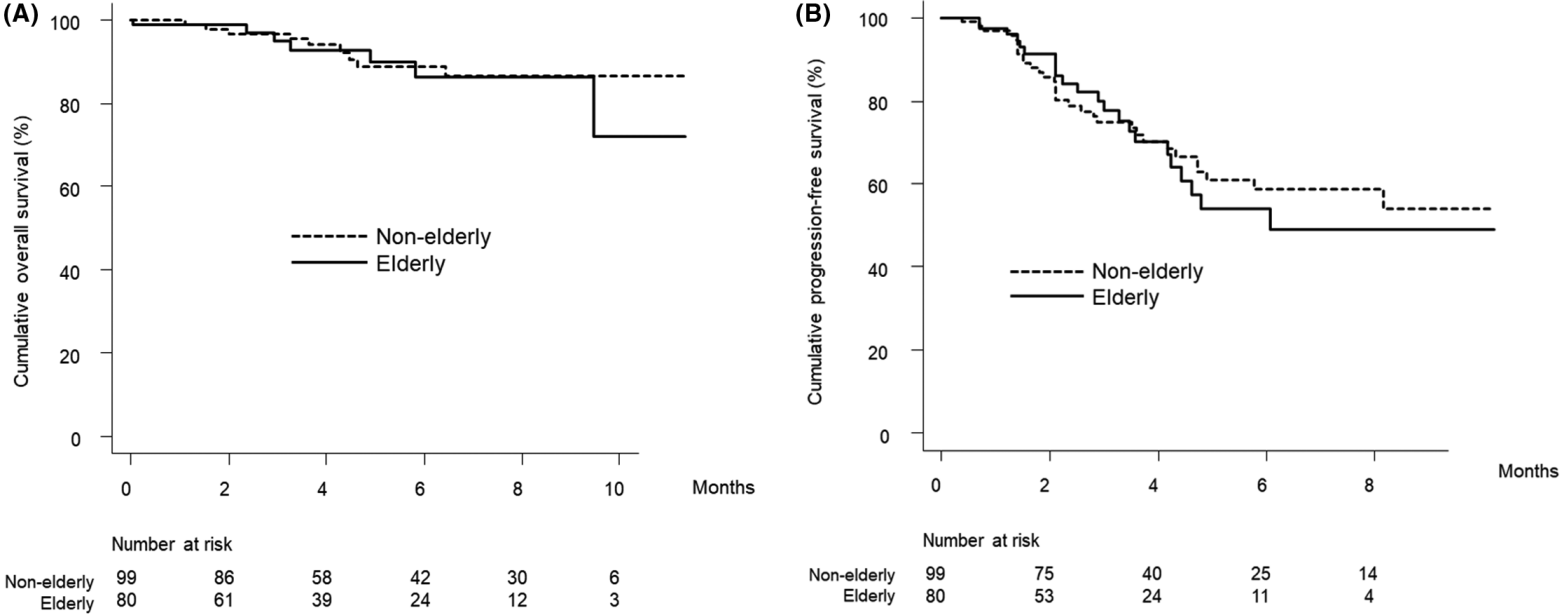
(A) Overall survival stratified by elderly status in patients who received Atez/Bev as first‐line systematic therapy. Cumulative overall survival at 3, 6, and 9 months was 94.9%, 86.4%, and 86.4% among elderly patients (solid line) and 96.6%, 88.9%, and 86.6% among non‐elderly patients (dotted line), respectively (*p* = 0.728). (B) Progression‐free survival stratified by elderly status in patients who received Atez/Bev as first‐line systematic therapy. Cumulative progression‐free survival at 3 and 6 months was 77.6% and 54.0% among elderly patients (solid line) and 74.9% and 58.8% among non‐elderly patients (dotted line), respectively (*p* = 0.805). Atez/Bev, atezolizumab plus bevacizumab

Figure 4A,B show the curves for overall and progression‐free survival stratified by elderly status. There were no significant differences in the overall or cumulative progression‐free survival curves for elderly versus non‐elderly patients (*p* = 0.728 and 0.805, respectively).

Table 6 shows the distribution of therapeutic response in this subgroup stratified by elderly status. There were no significant differences in therapeutic responses between elderly and non‐elderly patients.

**TABLE 6:** Therapeutic response in patients who received Atez/Bev as first‐line systemic therapy

	Elderly patients (*n* = 80)	Non‐elderly patients (*n* = 99)	*p*value
Complete response	5 (8.2%)	4 (4.4%)	0.325
Partial response	18 (29.5%)	18 (19.8%)	
Stable disease	29 (47.5%)	50 (54.9%)	
Progressive disease	9 (14.8%)	9 (14.8%)	
Not evaluated	19	18	
Overall response rate	24.2%	37.7%	0.102
Disease control rate	79.1%	85.2%	0.398

Abbreviation: Atez/Bev, atezolizumab plus bevacizumab.

Table 7 lists the treatment‐related adverse events that occurred in this subgroup. There were no significant differences in treatment‐related adverse events between elderly and non‐elderly patients, except for any grade of decreased appetite and grade ≥3 hypertension.

**TABLE 7:** Adverse events in patients treated with Atez/Bev as first‐line systemic therapy

	Elderly patients (*n* = 80)	Non‐elderly patients (*n* = 99)	*p*value
Liver injury			
Any grade	4 (5.0%)	14 (14.1%)	0.048
Grade ≥3	0 (0.0%)	3 (3.0%)	0.254
Fatigue			
Any grade	22 (27.5%)	24 (24.2%)	0.731
Grade ≥3	2 (2.5%)	0 (0.0%)	0.198
Decreased appetite			
Any grade	18 (22.5%)	18 (18.2%)	0.574
Grade ≥3	4 (5.0%)	0 (0.0%)	0.038
Proteinuria			
Any grade	18 (22.5%)	17 (17.2%)	0.449
Grade ≥3	6 (7.5%)	5 (5.1%)	0.554
Hypertension			
Any grade	12 (15.0%)	19 (19.2%)	0.553
Grade ≥3	6 (7.5%)	1 (1.0%)	0.046
Hypothyroidism			
Any grade	4 (5.0%)	8 (8.1%)	0.552
Grade ≥3	1 (1.2%)	1 (1.0%)	1.000
Fever			
Any grade	3 (3.8%)	9 (9.1%)	0.230
Grade ≥3	1 (1.2%)	2 (2.0%)	1.000
Other			
Any grade	31 (38.8%)	32 (32.3%)	0.432
Grade ≥3	6 (7.5%)	4 (4.0%)	0.346

Abbreviation: Atez/Bev, atezolizumab plus bevacizumab.

## 4 DISCUSSION

In this multicenter study with a large number of patients with unresectable HCC with Atez/Bev, there were no significant differences in the frequency of adverse events associated with Atez/Bev between the elderly and non‐elderly groups. In addition, the elderly and non‐elderly groups had similar overall and progression‐free survival before and after IPW adjustment for sex, ECOG‐PS, Child‐Pugh classification, HCC etiology, BCLC classification, and history of systematic therapy. Furthermore, in the subgroup analysis including only elderly patients, there were no differences in overall or progression‐free survival by age group (i.e., 75–79 years, 80–84 years, and 85 years or over). In this sub‐analysis of the elderly group, there were no significant differences in treatment‐related adverse events among these three age groups except for proteinuria of any grade. These results of those investigations suggest that Atez/Bev can be used efficaciously and safely in spite of age in patients with unresectable HCC.

Between 1990 and 2015, the incidence of liver cancer increased by 75% worldwide [25]. These dynamics reportedly reflect changes in population age distribution, population growth, age‐standardized incidence rates, and etiology [26]. During this period, a significant increase in HCC age‐standardized incidence due to hepatitis C virus was observed (+15.7%), while hepatitis B virus–related HCC significantly decreased (−18.9%) and no significant changes were observed for HCC due to alcohol (+13.5%) or other causes (−12.3%) [26]. Despite a decrease in age‐standardized incidence rates for HCC related to hepatitis B virus and other causes, the overall incidence of HCC has increased because of demographic changes, namely population growth and aging [26]. Kumada et al. [27] assessed the changes in patient characteristics over a 30‐year period using clinical data from 2347 patients at the time of initial HCC diagnosis. In their report [27], age at HCC diagnosis during the periods of 1990–1994, 1995–1999, 2000–2004, 2005–2009, 2010–2014, and 2015–2018 significantly increased: 63 (57–59), 65 (60–70), 68 (62–74), 70 (63–76), and 72 (65–78), and 74 (67–80) years, respectively (*p* < 0.001). Increased, from 63 (57–59) to 74 (67–80) years suggests that a quarter of patients who recently received a diagnosis of HCC were over 80 years old. In addition, Kudo [11] reported that the median overall survival for patients with all BCLC stages of HCC registered in Japan's nationwide follow‐up survey during the periods 1978–1980, 1981–1985, 1986–1990, 1991–1995, 1996–2000, 2001–2005, and 2006–2009 improved from 4 to 60 months, respectively. These findings strongly suggest that we frequently treat elderly patients with HCC who need various treatments, including systematic therapy, in clinical practice. As a result, it seems important to verify the safety and efficacy of new treatments for HCC in elderly patients using real‐world clinical data. Therefore, in this study, we clarified the safety and efficacy of Atez/Bev in elderly patients with unresectable HCC using clinical data from multiple centers.

The phase 3 IMbrave150 study [12] included 336 patients treated with Atez/Bev. The median age was 64 (56–71) years. With regard to safety in the IMbrave150 study [12], grade ≥3 adverse events related to Atez/Bev therapy occurred at a higher frequency in the sorafenib therapy group (46%, 71/156) than in the Atez/Bev therapy group (36%, 117/329). Among treatment‐related adverse events occurring at a rate ≥ 10%, adverse events that commonly occurred in the sorafenib therapy group, such as decreased appetite, hand–foot skin reaction, hypertension, and diarrhea, were uncommon in the Atez/Bev therapy group. Proteinuria and liver injury were slightly higher frequency in the Atez/Bev therapy group than in the sorafenib therapy group, although these adverse events were grade ≤2. In this study, the 317 patients treated with Atez/Bev had a median age of 74 (68–80) years. Namely, our cohort was 10 years older than the cohort in the phase 3 study. In the present study, any grade of liver injury, decreased appetite, fatigue, proteinuria, and hypertension occurred in ≥10% of patients. In addition, we clarified that there were no significant differences in the frequency of adverse events of any grade or grade ≥3 related to Atez/Bev between the elderly and non‐elderly groups. Regarding the subgroup analysis of patients treated with Atez/Bev as first‐line systemic therapy, we clarified that there were no significant differences in the frequency of adverse events associated with Atez/Bev between the elderly and non‐elderly groups, other than liver injury of any grade and grade ≥3 decreased appetite and hypertension. However, the frequency of grade ≥3 decreased appetite and hypertension in elderly patients was only 5.0% and 7.5%, respectively. In addition, the frequency of liver injury of any grade in elderly patients was lower than in non‐elderly patients. One advantage of the current study was having almost the same number of patients with unresectable HCC treated with Atez/Bev as the Atez/Bev group in the IMbrave150 study. Another advantage of the current study is that a quarter of patients were over 80 years old, which is more clinically relevant for patients in Japan. However, because of the risk of developing immune‐related adverse events in the non‐elderly as well as elderly patients with a history of auto‐immune disease, it was considered advisable not to offer Atez/Bev therapy to these patients. In fact, in this study, patients with a known history of auto‐immune disease were not treated with Atez/Bev.

In this study, BCLC classification at the start of Atez/Bev differed significantly between the elderly and non‐elderly groups, raising concerns regarding lead‐time bias. Therefore, BCLC classification was also used to adjust for IPW.

This study has several limitations. First, this study included its relatively short follow‐up period and retrospective nature. Although the present study included a large number of patients with unresectable HCC from multiple liver centers in Japan, further prospective validation studies with long‐term follow‐up period are warranted. The second limitation of the present study was that treatment of HCC including systemic chemotherapy and conversion therapy after Atez/Bev therapy was not investigated. Since treatment after Atez/Bev therapy might have an effect upon prognosis, further studies that include an investigation of HCC treatment after Atez/Bev therapy are also warranted. Furthermore, covariates that were likely to be associated with the prognosis of patients with HCC were used in calculating the propensity score. However, age is a factor that generally has a significant impact on the prognosis of patients with HCC. Therefore, it is necessary to consider selection bias in this study that the prognoses of the elderly and non‐elderly were comparable. In addition, unmeasured confounders were also considered one of the limitations of this study.

In conclusion, Atez/Bev can be used safely and efficaciously in spite of age in patients with unresectable HCC. Additionally, the safety and efficacy of Atez/Bev in patients treated with Atez/Bev as first‐line systemic therapy and patients who received Atez/Bev at any time were comparable. Further studies are warranted to confirm these findings in other populations.

## CONFLICT OF INTEREST

Atsushi Hiraoka: lecture fees from Bayer, Eisai, Eli Lilly, Otsuka. Takashi Kumada: lecture fees from Eisai. None of the other authors have potential conflicts of interest to declare.

## AUTHOR CONTRIBUTIONS

T Tada, T Kumada, and A Hiraoka conceived the research and participated in its design and coordination. T Tada, T Kumada, M Hirooka, A Hiraoka, K Kariyama, M Atsukawa, J Tani, KT Takaguchi, S Fukunishi, E Itobayashi, T Ishikawa, K Tsuji, H Ochi, KT Tajiri, H Toyoda, S Yasuda, T Nishimura, C Ogawa, T Hatanaka, S Kakizaki, N Shimada, K Kawata, T Tanaka, H Ohama, K Nouso, A Tsutsui, T Nagano, A Morishita, N Itokawa, T Arai, M Imai, T Okubo, Y Koizumi, A Naganuma, K Joko, S Nakamura, H Iijima, and Y Hiasa performed data curation. T Tada carried out statistical analyses and interpretation. T Tada, H Toyoda, T Hatanaka, A Hiraoka, and T Kumada drafted the manuscript. All authors have read and approved the final version of the manuscript

## Ethics Approval

The protocol used in the present study was approved by the Institutional Ethics Committee of Ehime Prefectural Central Hospital (IRB No. 30–66), based on the Guidelines for Clinical Research issued by the Ministry of Health and Welfare of Japan. All methods were carried out in accordance with relevant guidelines and regulations.

## Consent to participate

Written informed consent was obtained from each patient.

## Consent for publication

Written informed consent was obtained from each patient.

## Supporting information


Table S1

## Data Availability

The datasets are available from the corresponding author on reasonable request.
