# Supplementary material for: Safety and efficacy of atezolizumab plus bevacizumab in elderly patients with hepatocellular carcinoma: A multicenter analysis
Source: Cancer Med. 2022 Apr 19;11(20):3796–808. doi: 10.1002/cam4.4763 (PMC9582693; doi:10.1002/cam4.4763)
Supplement: Supplementary file 1 — Table S1 [file CAM4-11-3796-s001.pdf]

Supplementary table 1. Characteristics of patients who received Atez/Bev as first-line systemic therapy (n=179)

	Elderly patients (n=80)	Non-elderly patients (n=99)	p value
Age* (years)	81.0 (77.0–84.0)	68.0 (62.0–71.0)	<0.001
Sex (female/male)	26/54	15/84	0.007
ECOG-PS (0/1/2/3/4)	58/17/4/1/0	85/9/4/0/1	0.061
Body mass index (kg/m^2)	23.3 (21.1–25.3)	23.9 (21.3–26.8)	0.146
Etiology of HCC (hepatitis B/C/non-B, non-C)	7/34/39	21/28/50	0.031
Aspartate aminotransferase (IU/L)	36 (27–57)	36 (26–58)	0.979
Alanine aminotransferase (IU/L)	26 (17–38)	31 (20–43)	0.081
Albumin (g/dL)*	3.8 (3.5–4.1)	3.8 (3.4–4.1)	0.810
Total bilirubin (mg/dL)*	0.7 (0.6–1.0)	0.8 (0.6–1.1)	0.417
Platelet count ($\times 10^3$/m^3) *	13.0 (10.7–17.3)	13.0 (9.8–20.3)	0.943
Prothrombin time (%)*	88 (83–98)	89 (78–97)	0.229
Estimated glomerular filtration rate (mL/min/1.73 m^2)*	58.1 (47.2–72.2)	72.5 (59.0–83.8)	<0.001
α-fetoprotein (ng/mL)*	36.6 (7.2–542.0)	20.0 (5.7–293.8)	0.633
Child-Pugh class (A/B/C)	76/4/0	92/6/1	1.000
BCLC stage (A/B/C/D)	10/37/31/2	8/35/54/2	0.190
Follow-up duration* (months)	3.8 (2.0–6.7)	5.3 (3.1–8.2)	0.028
Deaths	7	9	1.000
Causes of death (liver-related/ non–liver-related diseases)	5/2	9/0	0.175

*Data are expressed as medians (interquartile range).

Atez/Bev, atezolizumab plus bevacizumab; ECOG-PS, Eastern Cooperative Oncology Group Performance Status; HCC, hepatocellular carcinoma; BCLC, Barcelona Clinic Liver Cancer.
